# Effectiveness of Adaptive E-Learning Environments on Knowledge, Competence, and Behavior in Health Professionals and Students: Protocol for a Systematic Review and Meta-Analysis

**DOI:** 10.2196/resprot.8085

**Published:** 2017-07-05

**Authors:** Guillaume Fontaine, Sylvie Cossette, Marc-André Maheu-Cadotte, Tanya Mailhot, Marie-France Deschênes, Gabrielle Mathieu-Dupuis

**Affiliations:** ^1^ Montreal Heart Institute Research Center Montreal, QC Canada; ^2^ Faculty of Nursing Université de Montréal Montreal, QC Canada; ^3^ Faculty of Medicine Université de Montréal Montreal, QC Canada; ^4^ Center for Innovation in Nursing Education Université de Montréal Montreal, QC Canada; ^5^ School of Librarianship and Information Science Université de Montréal Montreal, QC Canada

**Keywords:** adaptive learning environments, intelligent tutoring systems, interactive learning environments, medical education, nursing education, e-learning, systematic review, meta-analysis

## Abstract

**Background:**

Adaptive e-learning environments (AEEs) can provide tailored instruction by adapting content, navigation, presentation, multimedia, and tools to each user’s navigation behavior, individual objectives, knowledge, and preferences. AEEs can have various levels of complexity, ranging from systems using a simple adaptive functionality to systems using artificial intelligence. While AEEs are promising, their effectiveness for the education of health professionals and health professions students remains unclear.

**Objective:**

The purpose of this systematic review is to assess the effectiveness of AEEs in improving knowledge, competence, and behavior in health professionals and students.

**Methods:**

We will follow the Cochrane Collaboration and the Effective Practice and Organisation of Care (EPOC) Group guidelines on systematic review methodology. A systematic search of the literature will be conducted in 6 bibliographic databases (CINAHL, EMBASE, ERIC, PsycINFO, PubMed, and Web of Science) using the concepts “adaptive e-learning environments,” “health professionals/students,” and “effects on knowledge/skills/behavior.” We will include randomized and nonrandomized controlled trials, in addition to controlled before-after, interrupted time series, and repeated measures studies published between 2005 and 2017. The title and the abstract of each study followed by a full-text assessment of potentially eligible studies will be independently screened by 2 review authors. Using the EPOC extraction form, 1 review author will conduct data extraction and a second author will validate the data extraction. The methodological quality of included studies will be independently assessed by 2 review authors using the EPOC risk of bias criteria. Included studies will be synthesized by a descriptive analysis. Where appropriate, data will be pooled using meta-analysis by applying the RevMan software version 5.1, considering the heterogeneity of studies.

**Results:**

The review is in progress. We plan to submit the results in the beginning of 2018.

**Conclusions:**

Providing tailored instruction to health professionals and students is a priority in order to optimize learning and clinical outcomes. This systematic review will synthesize the best available evidence regarding the effectiveness of AEEs in improving knowledge, competence, and behavior in health professionals and students. It will provide guidance to policy makers, hospital managers, and researchers in terms of AEE development, implementation, and evaluation in health care.

**Trial Registration:**

PROSPERO International Prospective Register of Systematic Reviews: CRD42017065585; https://www.crd.york.ac.uk/PROSPERO/display_record.asp?ID=CRD42017065585 (Archived by WebCite® at http://www.webcitation.org/6rXGdDwf4)

## Introduction

### Background

Since the beginning of the 21^st^ century, the complexification of care and the limitation of financial resources in health systems across the globe pose great challenges to the education of health professionals [1]. Academic and clinical leaders must find innovative and effective ways to maintain and update the curricula of their institutions in order to address systemic problems, such as the mismatch of health professionals’ competencies to patient and population needs [1]. We have known since the 1980s that individual learning, through one-on-one human tutoring, is more effective than learning through group lectures [2,3]. The consideration of users’ navigation behavior, individual goals, knowledge, and preferences provides opportunities for individualized instruction and optimizes learning outcomes [4]. However, while one-on-one human tutoring has its benefits, it is costly, lacks accessibility, and realistically cannot be replicated on a large scale.

E-learning, defined as instruction delivered on a digital device that is intended to support learning [5], is an ever-expanding field in health sciences education. Conventional e-learning courses use words, in the form of spoken or printed text, and multimedia (eg, illustrations, animations, videos) [5]. Content is typically presented linearly, much like reading a book. E-learning can be both asynchronous, (i.e., being designed for self-study) and synchronous (i.e., attending a Web-based class taught by an instructor in real time) [5]. Various presentations of e-learning are increasingly present in clinical settings for the continuing education of health professionals [6] and in academic settings for the education of health professions students [7]. Systematic reviews have demonstrated the effectiveness of e-learning to optimize knowledge, competence, and behavior in health professionals and students [8-13]. E-learning is generally considered to be as effective as non–e-learning educational interventions, such as traditional classroom instruction and printed text in improving learning outcomes [8,12]. Specific instructional design variations within e-learning platforms, such as interactivity, feedback, repetition, and practice exercises, result in better learning outcomes [14].

While e-learning can provide these features, it generally fails to provide individualized instruction equivalent to one-on-one human tutoring [15,16]. Indeed, e-learning is rarely designed to suit the learning goals, knowledge, and preferences of health professionals and students. Moreover, e-learning doesn’t provide opportunities for case-based problem solving and simulations of complex real-world tasks while providing tailored feedback and guidance [15]. This is problematic because these instructional strategies have been shown to be effective to improve learning outcomes in face-to-face education [17-19].

Adaptive e-learning environments (AEEs) shows great promise in providing tailored instruction to health professionals and students by adapting the training to each user [20]. By continuously collecting data to build a user’s profile (eg, navigation behavior, individual objectives, knowledge, preferences), interpreting these data through algorithms, and adapting in real-time content, navigation, presentation, multimedia, and tools, AEEs can provide a dynamic and evolutionary learning path for each user [21]. However, a wide variety of systems may provide adaptive functionality with various levels of complexity in different fields of study. For instance, adaptive hypermedia systems [21-23] and intelligent tutoring systems [24-26] may both provide variations of an adaptive functionality.

In recent years, AEEs with various levels of complexity, ranging from systems using an adaptive functionality, to systems using artificial intelligence, have been evaluated in some academic settings with positive results regarding learning outcomes [24-27]. However, the effectiveness of AEEs for health professionals’ and students’ education remains unclear. To our knowledge, no systematic review assessed the effectiveness of AEEs on knowledge, competence, and behavior in health professionals and students.

### Systematic Review Objective

To systematically identify, appraise, and synthesize the best available evidence regarding the effectiveness of AEEs in improving knowledge, competence, and behavior in health professionals and health professions students.

### Systematic Review Question

What is the effectiveness of AEEs in improving knowledge, competence, and behavior in health professionals and students in comparison with nonadaptive e-learning environments or non–e-learning educational interventions?

## Methods

### Systematic Review Protocol Development and Registration

This systematic review protocol has been developed according to the Preferred Reporting Items for Systematic review and Meta-Analysis Protocols (PRISMA-P) [28] (Multimedia Appendix A).

This protocol has also been registered prospectively on the PROSPERO database (registration number: CRD42017065585).

### Inclusion Criteria

#### Types of Studies

We will include randomized controlled trials (RCTs), non-RCTs, controlled before-after studies, interrupted time series studies, and repeated measures studies. In order to assess the eligibility of study designs, we will use the algorithm proposed by the Effective Practice and Organisation of Care (EPOC) Cochrane Review Group [29]. If no studies employing these designs are identified, we will consider quasiexperimental study designs for inclusion, such as 1-group pretest/posttest studies and nonequivalent groups studies.

Studies published in French or English, between 2005 and 2017, and conducted in all academic and clinical settings will be considered for inclusion, regardless of the geographic location.

A meta-analysis on the effectiveness of a subtype of AEEs, intelligent tutoring systems, with college students in computer science, physics, and mathematics, found that studies published before 2005-2006 seem to have a bias toward more positive results [25]. This could be explained by the novelty of e-learning in earlier studies, which could have positively affected student motivation and learning outcomes. Thus, we will only include primary studies published from 2005 onward.

#### Types of Participants

We will consider primary studies conducted with licensed health professionals, students, trainees, and residents in any health care context. From now on, in the context of this review, we will call health professionals and students “users.”

#### Types of Interventions

For the purpose of this review, we define an AEE as an e-learning platform, which continuously collects data to build each user’s profile (eg, navigation behavior, individual objectives, preferences, knowledge), interprets these data through algorithms, and adapts in real-time the content (eg, showing/hiding information, providing tailored feedback), navigation (eg, specific links and paths), presentation (eg, device adaptation, page layout), multimedia presentation (eg, images, models, views, widgets, graphics items, scripts, and strategies), or tools (eg, different set of features for the different types of users) to provide a dynamic and evolutionary learning path for each user [20,21,30]. We will use the definitions of each type of adaptation proposed by Knutov and colleagues [21]. The AEE can involve variable levels of technological complexity, ranging from simple adaptive functionality to the use of artificial intelligence [20].

#### Types of Comparators

Eligible comparators will be nonadaptive e-learning interventions and non–e-learning educational interventions. Nonadaptive e-learning interventions can include interactive features, such as quizzes and practice exercises, and multimedia, but present the same content linearly for each user. Non–e-learning educational interventions can be, for example, traditional classroom instruction, a PowerPoint presentation, printed text, or a combination of these interventions.

#### Types of Outcomes Measures

We will consider for inclusion studies reporting the following outcomes: knowledge, competence, and behavior in health professionals and students.

**Figure 1:**
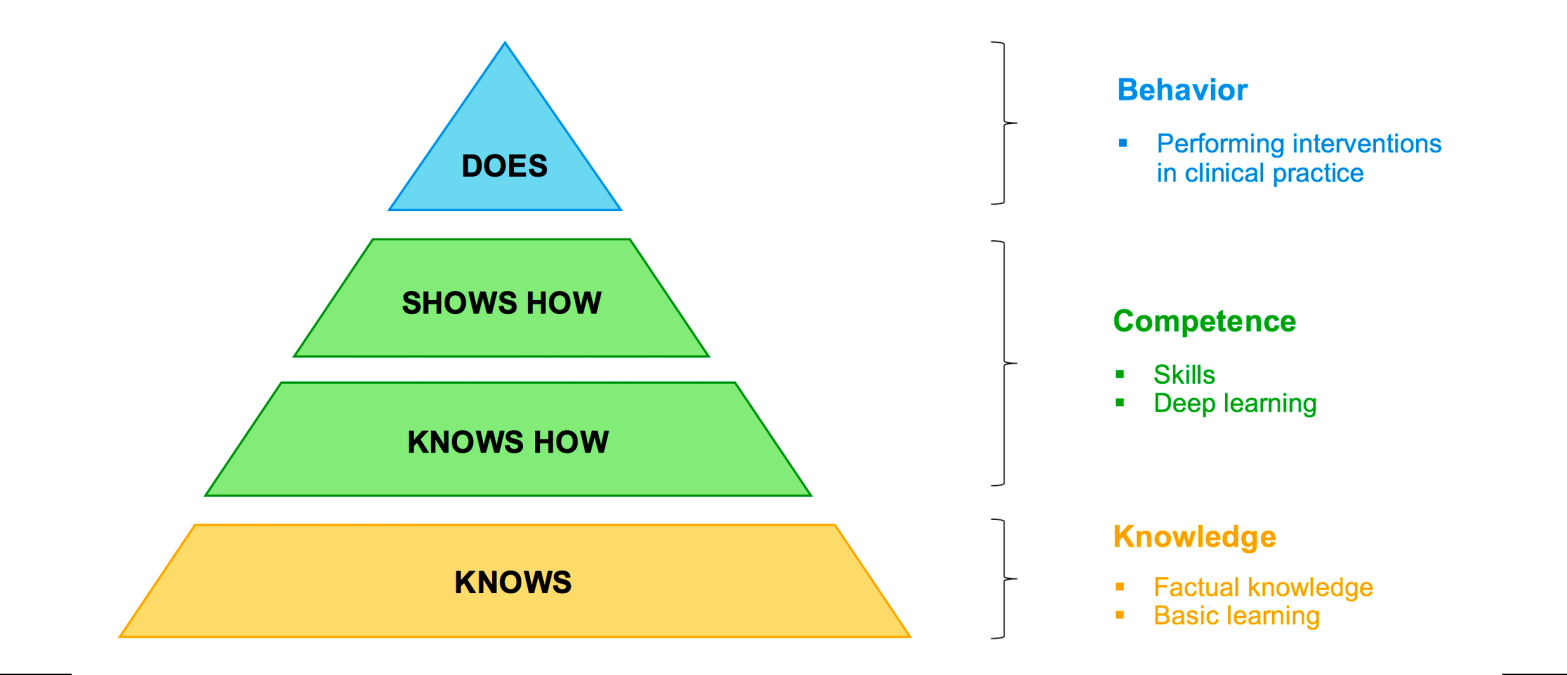
The modified conceptual model of Miller.

In order to define the outcomes of this systematic review, we adopted the modified conceptual model of Miller [31], which is a framework that identifies 4 stages of clinical practice development: knows, knows how, shows how, and does. Through cognitive and behavioral changes, health professionals and students progress from developing their knowledge about a particular health condition to performing interventions in clinical practice [31]. In the modified version of the model [32], the stages of development have been modified to better match the design and effects of educational interventions (Figure 1).

##### Primary Outcome Measures

Primary studies reporting an objective measure of users’ knowledge (eg, multiple-choice test for assessing factual or conceptual understanding) or a subjective measure of users’ knowledge (eg, self-reported knowledge) will be considered for inclusion.

##### Secondary Outcome Measures

Primary studies reporting an objective measure of users’ competence (eg, behavior change counseling competence scores) or a subjective measure of users’ competence (eg, self-reported skills) will be considered for inclusion.

Primary studies reporting an objective measure of users’ behavior (eg, clinical interventions reported in patients’ medical file, number of tests ordered) and a subjective measure of users’ clinical behavior (eg, self-reported performance of clinical interventions) will be considered for inclusion.

### Exclusion Criteria

Any study that does not correspond to the inclusion criteria in terms of study design, participants, intervention, comparator, or outcome measures will be excluded.

We will also exclude: (1) studies that do not provide a sufficient description of the AEE assessed for judging which type(s) and techniques of adaptation are applied; (2) RCTs not published in peer-reviewed journals (eg, dissertations and case reports); (3) systematic reviews, literature reviews, letters, commentaries, editorials, study protocols; and (4) secondary subgroup analyses of RCTs or RCT-generated data modeling studies.

### Literature Search

#### Information Sources

##### Bibliographical Databases

Eligible primary studies will be identified through a comprehensive literature search of 6 bibliographic databases: CINAHL (EBSCO), EMBASE (OVID), ERIC (ProQuest), PsycINFO (APA PsycNET), PubMed (NCBI), and Web of Science – SCI and SSCI (ISI – Thomson Scientific).

##### Hand Searching

Relevant journals will be hand-searched for additional articles. Such journals include: *User Modeling and User-Adapted Interaction*, *Computers and Education*, *Journal of Computer Assisted Learning*, *Journal of Medical Internet Research*, *Educational Technology Research and Development*, and *British Journal of Educational Technology*.

##### Reference Searching

Reference lists of primary studies included will be hand-searched for additional relevant articles. We will also search the reference lists of systematic reviews and meta-analyses in the field of AEEs for relevant articles.

### Search Strategy for Bibliographical Databases

The search strategy was developed in collaboration with a Master’s student in information science. The search strategy uses a combination of keywords and MeSH terms that relate to 3 key concepts (adaptive e-learning environments; health professionals/health sciences students; effects on knowledge, skills, and behavior) (Multimedia Appendix B). We first developed the strategy for PubMed, and then translated it for other databases (Multimedia Appendix C).

### Data Collection and Analysis

#### Selection of Studies

The titles and abstracts of studies retrieved by the search strategy will be independently screened and “eligibility criteria will be applied by 2 review authors. Disagreements will be resolved through discussion and consensus. A third author will be involved in case of a persistent disagreement. Reference management will be done using the EndNote software, version 8.0.

A full-text assessment of selected articles after the initial screening will be independently conducted by 2 review authors. Access to articles will be gained through the library system of the Université de Montréal. Reasons for the exclusion of articles will be documented and the process of study selection will be reported in a PRISMA flow diagram [33].

#### Data Extraction and Management

The data of included studies will be extracted using a modified version of the data collection form of the EPOC Cochrane Review Group data collection checklist by 2 review authors [34] (Textbox 1). Data will then be entered in the Review Manager (RevMan) software, version 5.1. In the case of unclear data, authors will be contacted to obtain relevant data. Data collection forms will then be sent to the first authors of included primary studies for validation.

#### Assessment of Risk of Bias

The quality of included studies will be independently assessed by 2 review authors using the EPOC risk of bias criteria based upon the data extracted with the data collection checklist [34]. Discrepancies in rating will be resolved through discussion and consensus. Based on the EPOC Cochrane Review Group risk of bias criteria [34], the following 9 criteria will be considered to assess included studies for potential bias: (1) Was the allocation sequence adequately generated? (2) Was the allocation adequately concealed? (3) Were baseline outcome measurements similar? (4) Were baseline characteristics similar? (5) Were incomplete outcome data adequately addressed? (6) Was knowledge of the allocated interventions adequately prevented during the study? (7) Was the study adequately protected against contamination? (8) Was the study free from selective outcome reporting? (9) Was the study free from other risks of bias?

Each criterion will be rated as “low risk” if the bias is unlikely to have seriously affected the results, “high risk” if the bias likely weakened the reliability of the results, and “unclear risk” when there is not enough information to rate the bias as low or high [35]. Justification of each author’s assessment will be noted in the risk of bias table.

**Information to extract from included primary studies.**

Population and setting

- For descriptive purposes: study setting, inclusion criteria, and exclusion criteria.
- For statistical analyses purposes: study population and study sample.

Methods

For descriptive purposes: study aim, study design, unit of allocation, study start and end date, duration of participation.

Risk of bias assessment

For statistical analysis purposes: random sequence generation, allocation concealment, similarity of baseline outcome measurements, similarity of baseline characteristics, incomplete outcome data, blinding of participants and personnel, blinding of outcome assessments, measures against contamination, selective outcome reporting, other risk of bias.

Participants

For descriptive purposes: withdrawals and exclusions, age, sex, level of instruction, number of years of experience as a health professional, practice setting, previous experience using e-learning.

Interventions (AEE and comparator)

- For descriptive purposes: name of intervention, theoretical framework, statistical model/algorithm, subject, number of training sessions, duration of each training session, mode of delivery, presence of other educational interventions and strategies.
- For statistical analysis purposes: total duration of the training, type and degree of adaptation within the AEE (content, navigation, presentation, multimedia presentation, tools).

Outcomes

For descriptive purposes: name, time-points measured, definition, person measuring, unit of measurement, scales, validation of measurement tool.

Results

- For descriptive purposes: comparison, time-point, baseline data, statistical methods used, and key conclusions.
- For statistical analysis purposes: results according to our primary (knowledge) and secondary (competence, behavior) outcomes.

**Table 1:** Reasons for downgrading the quality of a body of evidence for a specific outcome.

Factor	Description	Interpretation
Limitations in the design and implementation (within-study risk of bias)	The assessments with the EPOC Cochrane Group risk of bias criteria should feed directly into this factor.	Review authors will interpret the risk of bias as follows: ‘low risk of bias’ would indicate ‘no limitation’; ‘unclear risk of bias’ would indicate either ‘no limitation’ or ‘serious limitation’; ‘high risk of bias’ would indicate either ‘serious limitation’ or ‘very serious limitation’.
Indirectness of evidence	Indirect comparisons between intervention A and B; Restricted version of the main review question in terms of population, intervention, comparator, or outcomes.	Review authors will make judgments based on differences in anticipated effects in the group of primary interest.
Unexplained heterogeneity or inconsistency of results	Studies yield widely differing estimates of effect (heterogeneity or variability in results)	Review authors will downgrade the quality of evidence when there is no plausible explanation to the heterogeneity that exists and affects the interpretation of results.
Imprecision of the results	Studies include few participants and few events and have wide confidence intervals.	Review authors will lower their rating of the quality of the evidence if there is imprecision in results of included primary studies.
High probability of reporting bias	The assessments made regarding funnel plot asymmetry should feed directly into this factor.	Review authors will downgrade the quality of evidence level if there is a high probability of reporting bias based on funnel plot asymmetry.

**Subgroup analysis.**

Change in targeted health professionals and students

- Doctors and student doctors;
- Nurses and student nurses;
- Other allied health professionals and students.
- Hypothesis: AEEs are more effective for doctors and student doctors than for nurses and student nurses or for health professionals and students.

Change in degree of AEE adaptation: we will rate the degree of adaptation of each AEE from 1-5 according to the types of adaptation (content, navigation, presentation, multimedia presentation, tools).

- ≤2 of 5 types of adaptation;
- ≥3 of 5 types of adaptation.
- Hypothesis: AEEs including ≥3 of 5 types of adaptation are more effective than those including ≤2 of 5 types of adaptation.

Change in type of AEE adaptation

- Content adaptation;
- Other types of adaptation (navigation, presentation, multimedia presentation, tools).
- Hypothesis: AEEs including content adaptation are more effective than those including other types of adaptation.

Change in AEE training program duration

- AEE training programs lasting 1 week or less;
- AEE training programs lasting more than 1 week.
- Hypothesis: AEEs training programs lasting more than 1 week are more effective than those lasting 1 week or less.

Change in publication years

- Studies published before 2010;
- Studies published after 2010.
- Hypothesis: AEEs reported in studies published after 2010 are more effective than those reported in studies published before 2010.

#### Dealing With Unclear Data

We will contact the investigators of included studies if there is any unclear or missing data. If unsuccessful, we will provide a narrative synthesis of the data as presented in the study.

#### Assessment of Heterogeneity

Heterogeneity will first be assessed by examining the characteristics of included studies, the similarities and disparities between the types of participants, the types of interventions, and the types of outcomes.

Heterogeneity will then be assessed by using the chi-square and the *I*^2^ statistics within the RevMan software. The *I*^2^ statistic describes the percentage of variance in effect estimates that is due to heterogeneity. As suggested by Higgins et al. [36], we will interpret the *I*^2^ values as follows: 0%-40%: might not be important; 30%-60%: may represent moderate heterogeneity; 50%-90%: may represent substantial heterogeneity; and 75%-100%: considerable heterogeneity.

We intend to use the random-effects model where moderate to substantial heterogeneity is observed. If substantial or considerable heterogeneity is observed and study effects are discordant, we will not pool data, unless heterogeneity is explained by subgroup differences.

#### Assessment of Reporting Biases

Reporting biases will be assessed by using funnel plots if more than 10 studies are included in the meta-analysis, as recommended by the Cochrane Handbook. We will follow the guidelines regarding funnel plot asymmetry as described in the *Cochrane Handbook for Systematic Reviews of Interventions* 5.1.0 [35]. For analyses performed on less than 10 primary studies, we will assess reporting bias qualitatively.

#### Assessment of the Quality of Evidence

The quality of the evidence regarding the outcomes reported in this systematic review will be assessed using the Grading of Recommendations Assessment, Development, and Evaluation (GRADE) approach, based upon the data extracted with the data collection checklist [37,38]. The GRADE approach specifies 4 levels of quality (high, moderate, low, and very low) for each individual outcome.

In order to attribute a level of quality, the GRADE approach considers 5 factors for downgrading the quality of a body of evidence for a specific outcome (Table 1). The certainty of the evidence will be independently assessed by 2 review authors. Any disagreement will be resolved through discussion.

#### Data Synthesis

Characteristics of included primary studies, such as population studied and study design, will be presented in a table format.

Data will then be synthesized by a descriptive analysis. We will describe the characteristics of AEEs including, when possible: intervention name, theoretical framework, statistical model or algorithm, subject, number of training sessions, duration of each training session, total duration of the training, mode of delivery, and presence of other educational interventions and strategies.

We will undertake a meta-analysis that will compare changes between intervention and control participants in primary and secondary outcomes, for which data from at least 2 studies are available. Knowledge, competence, and behavior will be addressed and analyzed separately. Statistical analysis will be conducted upon consideration of dichotomous outcome variables (eg, content adaptation, yes/no) and continuous outcome variables (eg, change in behavior change counseling competence scores).

#### Sensitivity Analysis

We will perform sensitivity analyses in order to exclude high risk of bias studies (as assessed with the EPOC risk of bias criteria) and small sample size studies (n≤20). Sensitivity analyses will allow us to determine if our conclusions are robust or if the key findings disappear with the exclusion of high risk of bias and small sample size studies.

#### Subgroup Analysis

Contextual heterogeneity will be considered by conducting the analyses in subgroups, according to potential effect modifiers. If sufficient data is available, we plan to perform 5 subgroup analyses (Textbox 2).

## Results

The review is in progress. We plan to submit the manuscript in the beginning of 2018. The anticipated findings of this systematic review will have implications for policy, practice, and research. First, it will provide evidence for policy makers and hospital managers of whether or not AEEs can increase the learning effectiveness and efficiency for health professionals and students, potentially lowering training costs and optimizing clinical practice. Second, this systematic review will identify specific considerations regarding AEE design, implementation, and evaluation in health care, indicating what would need to be taken into account for future studies.

## Discussion

Providing tailored instruction to health professionals and students is a priority in order to optimize learning and clinical outcomes. This systematic review will provide a summary of the best available evidence regarding the effectiveness of AEEs in improving the knowledge, competence, and behavior of health professionals and students.

## Appendix

Multimedia Appendix 1 PRISMA-P Checklist.

Multimedia Appendix 2 Concept plan used to build the search strategy.

Multimedia Appendix 3 Search strategy for PubMed.

## Abbreviations

AEE: adaptive e-learning environment
EPOC: effective practice and organisation of care
GRADE: grading of recommendations assessment, development and evaluation
PRISMA-P: preferred reporting items for systematic review and meta-analysis protocols
RCT: randomized control trial
