# Correction of: Effectiveness of Adaptive E-Learning Environments on Knowledge, Competence, and Behavior in Health Professionals and Students: Protocol for a Systematic Review and Meta-Analysis

**DOI:** 10.2196/resprot.8377

**Published:** 2017-08-15

**Authors:** Guillaume Fontaine, Sylvie Cossette, Marc-André Maheu-Cadotte, Tanya Mailhot, Marie-France Deschênes, Gabrielle Mathieu-Dupuis

**Affiliations:** ^1^ Montreal Heart Institute Research Center Montreal, QC Canada; ^2^ Faculty of Nursing Université de Montréal Montreal, QC Canada; ^3^ Faculty of Medicine Université de Montréal Montreal, QC Canada; ^4^ Center for Innovation in Nursing Education Université de Montréal Montreal, QC Canada; ^5^ School of Librarianship and Information Science Université de Montréal Montreal, QC Canada

In the paper by Fontaine et al, entitled “Effectiveness of Adaptive E-Learning Environments on Knowledge, Competence, and Behavior in Health Professionals and Students: Protocol for a Systematic Review and Meta-Analysis” [JMIR Res Protoc 2017;6(7):e128], a passage was inadvertently omitted during copyediting. Under the subheading, *Secondary Outcome Measures*, the following should have been included:

“Primary studies reporting an objective measure of users’ competence (eg, behavior change counseling competence scores) or a subjective measure of users’ competence (eg, self-reported skills) will be considered for inclusion.”

The paragraph given above should have preceded the one below:

“Primary studies reporting an objective measure of users’ behavior (eg, clinical interventions reported in patients’ medical file, number of tests ordered) and a subjective measure of users’ clinical behavior (eg, self-reported performance of clinical interventions) will be considered for inclusion.”

In addition, the trial registration number and associated url links were left out of the abstract. This information should have appeared as follows: *Trial Registration:* PROSPERO International Prospective Register of Systematic Reviews: CRD42017065585; https://www.crd.york.ac.uk/PROSPERO/ display_record.asp? ID=CRD42017065585 (Archived by WebCite® athttp://www.webcitation.org/6rXGdDwf4).

This omission has been corrected in the online version of the paper on the JMIR website on August 15, 2017, together with the publication of this correction notice. Because this was made after submission to PubMed, the correction notice has been submitted to PubMed. The corrected metadata have also been resubmitted to CrossRef.

