# Supplementary figures and images for: Effectiveness of Adaptive E-Learning Environments on Knowledge, Competence, and Behavior in Health Professionals and Students: Protocol for a Systematic Review and Meta-Analysis
Source: JMIR Res Protoc. 2017 Jul 5;6(7):e128. doi: 10.2196/resprot.8085 (PMC5517824; doi:10.2196/resprot.8085)

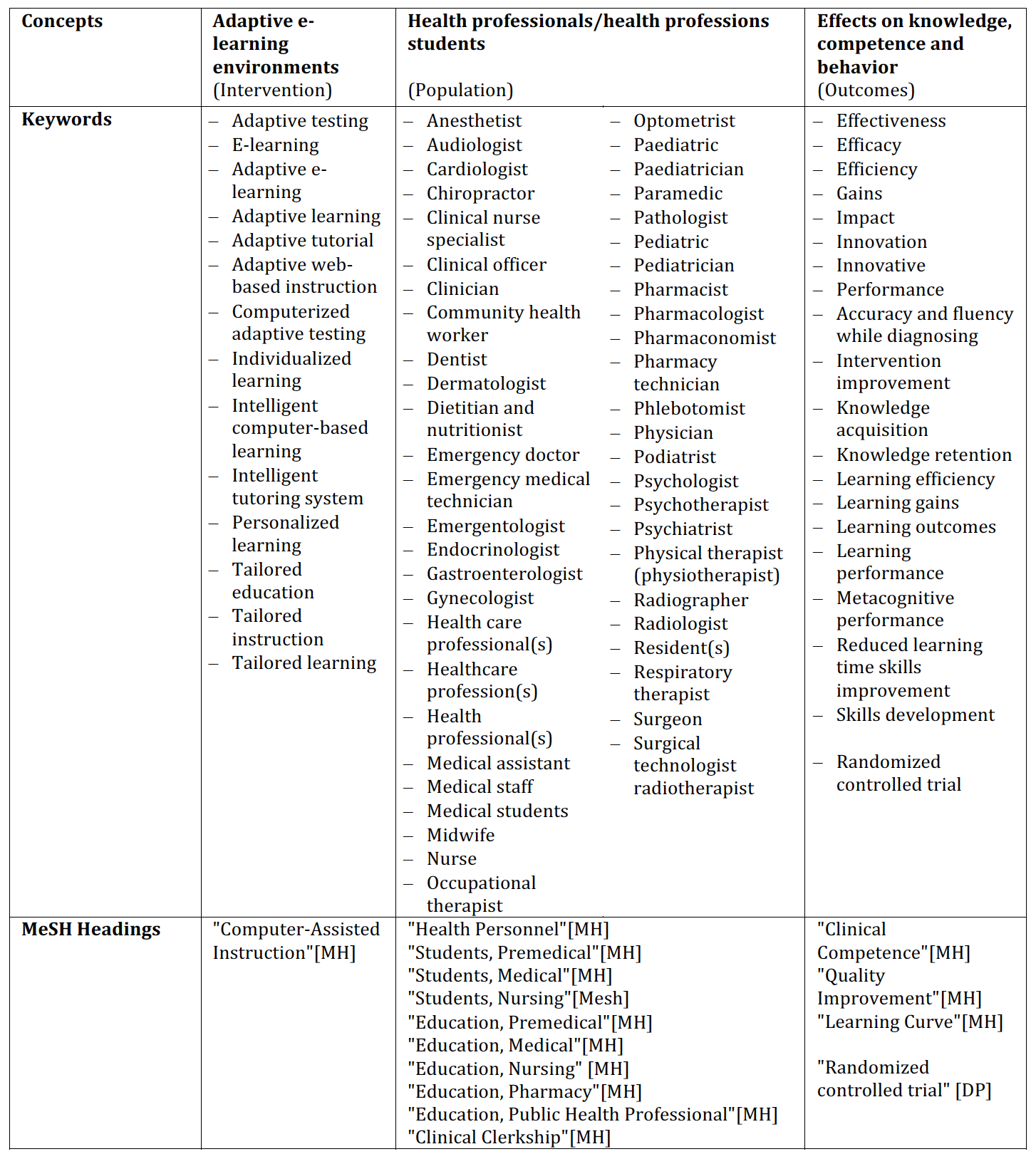

Supplement: Multimedia Appendix 2 [file resprot_v6i7e128_app2.png]

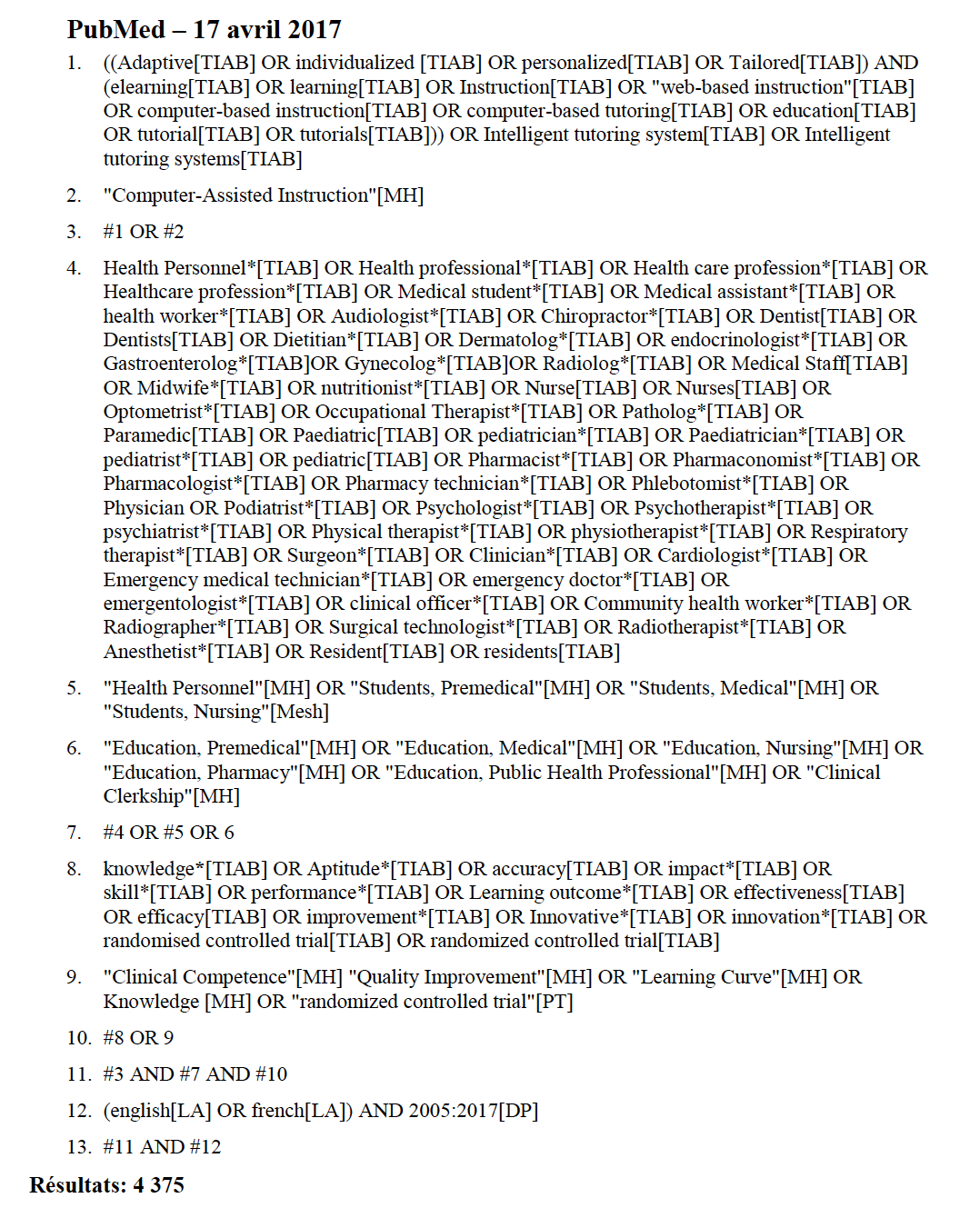

Supplement: Multimedia Appendix 3 [file resprot_v6i7e128_app3.png]
